# Reconstruction of soft tissue defects of hand: A systematic approach

**DOI:** 10.12669/pjms.40.1.7484

**Published:** 2024

**Authors:** Mansoor Khan, Waqas Hayat, Hidayat ullah, Nasir Hayat Khan

**Affiliations:** 1 Mansoor Khan, MBBS, FCPS Burns and Plastic Surgery Center, Hayatabad Medical Complex, Peshawar, Pakistan; 2 Waqas Hayat, MBBS, FCPS Burns and Plastic Surgery Center, Hayatabad Medical Complex, Peshawar, Pakistan; 3 Hidayat ullah, MBBS, FCPS Burns and Plastic Surgery Center, Hayatabad Medical Complex, Peshawar, Pakistan; 4 Nasir Hayat Khan, MBBS, FCPS Burns and Plastic Surgery Center, Hayatabad Medical Complex, Peshawar, Pakistan

**Keywords:** Hand, Hand injuries, Acquired hand deformities, Soft tissue injuries, Plastic surgery procedures, Hand reconstruction

## Abstract

**Background and Objective:**

A thorough insight into the management of hand injuries can shape the approach of a surgeon in order to achieve optimal outcomes for the patients. The aim of this study was to share our experience in reconstruction of the hand and establishing an algorithm for classification and treatment of hand injuries.

**Methods:**

This is a descriptive cross sectional study and was conducted from January 2020 to August 2022 at Burns and Plastic Surgery center, Peshawar. Data was collected from medical records about the patient demographics, mechanism of injury and type of procedures done. Defect size was classified into small (<5cm), medium (5cm to 10 cm) and large (>10cm). The defect site and size was cross tabulated against the method of soft tissue reconstruction in order to make the algorithm for reconstruction of hand injuries. Data was analyzed using SPSS.

**Results:**

The study population included 41 (75.9%) male and 13 (24.1%) female patients, mean age 31.56±14.1. Machine injuries (33.3%) and electric burns (24.1%) were the most common cause of hand soft tissue defects. The most commonly performed flap was Posterior introsseous artery (PIA) flap, followed by First dorsal metacarpal artery (FDMA) flap. Flap necrosis was observed in three cases (5.6%).

**Conclusion:**

This treatment algorithm for coverage of soft tissue defects in hand injuries will help with the decision making process of hand reconstruction and has didactic value for novice surgeons. It will also form the foundation for further work on this aspect of hand injuries.

## INTRODUCTION

Human hand is by far, the most important driving force for the technological growth of mankind. With growing population density and industrialization, injuries of hand has increased substantially. A study reported almost half of emergency visits consisted of hand injuries.1 Socio-demographic factors can also influence the incidence of hand injuries.2 Athletes and labor working in industry have disproportionately increased incidence of hand injuries.2,3 These injuries are often neglected in polytrauma patients as they are considered nonlife threatening.4 Prolonged recovery in hand injuries can reduce the health related quality of life (HRQoL) in patients.5,6 They can have significant impact on the rehabilitation and well-being of the patient in the long term.7,8

Patients with other associated injuries, low socio-economic class, female gender and people with suboptimal outcomes report a decrease in the quality of life.5,9 Recent studies have shown that there is a decrease in the number of surgeons who would respond to the emergency department calls related to hand surgery.10 As there is an increase in incidence of hand injuries presenting to the emergency departments, the low number of hand call surgeons can be a real issue for providing quality care to the patients.11,12 This can sometimes lead to delays in finding appropriate treatment for these patients.13,14

Appropriate intervention, when done in a timely fashion in hand injuries can lead to early recovery. This also positively impacts on the psychological aspects of injury by decreasing the duration of treatment, lessening the associated pain and improving the odds of return to work. A thorough insight into the management of hand injuries can shape the approach of a surgeon in order to achieve optimal outcomes for the patients. This will help not only in the standardization of the treatment options for hand surgery but will also make the treatment resource efficient. This study will have a didactive value for fellows training in the hand surgery. The aim of this study was to share our experience in reconstruction of the hand and establishing an algorithm for classification and treatment of hand injuries.

## METHODS

This descriptive cross-sectional study was conducted at Burns and Plastic Surgery Center, Peshawar from January 2020 to August 2022.

### Ethical Approval

The approval for this study was obtained from ethical board at Burns and Plastic Surgery Center (17/REB/B&PSC/22) on 9^th^ March, 2022. Informed consent for all photographs were taken from the patients beforehand. Patient files were reviewed and all patients who had undergone soft tissue reconstruction of the hand were included in this study. Data was collected about the patient demographics, mechanism of injury and type of procedures done. Defect size was classified into small (<5cm), medium (5cm to 10 cm) and large (>10cm). Involvement of structures such as tendons, neurovascular involvement and bony injuries was also documented. Data for reconstruction of soft tissues was also retrieved from the records. Data was analysed in SPSS. Frequencies and percentages were calculated for mechanism of injury, associated injuries and flaps performed. The defect site and size was cross tabulated against the method of soft tissue reconstruction in order to make the algorithm for reconstruction of hand injuries. Mechanism of injury was cross tabulated with against time taken to reconstruct.

## RESULTS

**Fig.1:**
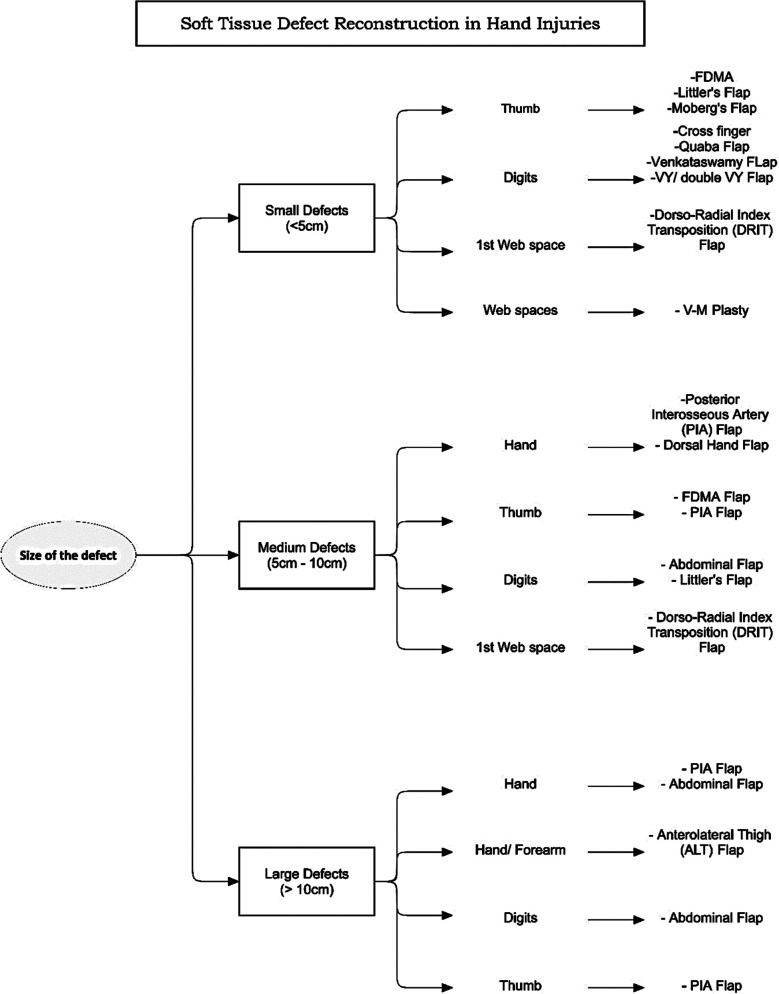
Algorithm for the soft tissue defects of hand. Defects classified according to size and region. Reconstructive options given for each defect.

The study population included 41 (75.9%) male and 13 (24.1%) female patients, having age ranging from five years to 65 years (M=31.56, SD =14.1). In 34 (63%) patients, right side was affected. Machine injuries were the most common cause of hand soft tissue defects (33.3%), followed by electric burns (24.1%). Other types of defects were secondary to blast injuries, RTAs, infection and post contracture release. The thumb (n=17, 31.5%), followed by other digits (n=12, 22.2%), were the most affected parts in our study population (Fig.1). Small defects were the most common (n=25, 46.3%) followed by medium and large defects. Electric burns (41.6%) and blast injuries (25%) were the main cause of large size defects. Out of the total, 53.7% patients had associated injuries (Table-I) and 59.3% had fractures of different bone of the hand.

**Table-I:** Associated neurovascular and tendon Injuries with soft tissue defects.

Associated injuries	Frequency	Percent
Nill	25	46.3
Tendon Injuries	13	24.1
Nerve Injuries	1	1.9
Amputation	9	16.7
Neurovascular, Tendons	2	3.7
Neurovascular	3	5.6
Nerves, Tendons	1	1.9

Total	54	100.0

**Fig.2:**
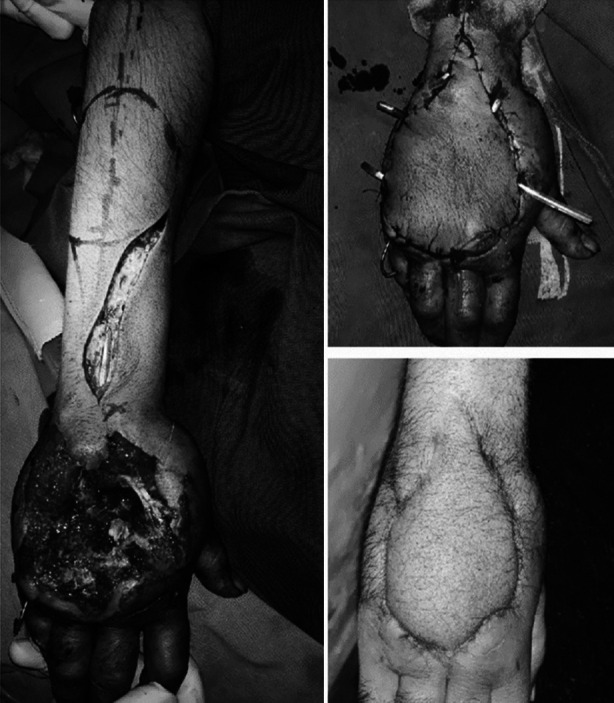
Figure shows machine injury to the dorsum of the hand. Markings for Posterior interosseous artery (PIA) flap visible in figure. Immediate and late post-operative picture of PIA flap. Tendon reconstruction done after attaining a supple soft tissue coverage.

Defects resulted from contracture release, malignancy excision and correction of congenital anomalies were reconstructed immediately. Defects resulting from electric burns, infection and blast injuries took longest to complete the reconstruction of soft tissues (Table-II). The flaps done to cover the defects are given in the form of algorithm as shown in Fig.1. The most common flap performed in our study population was Reverse Posterior Interosseous Artery (PIA) Flap (Fig.2) accounting for 29.6% (n=16), followed by First Dorsal Metacarpal Artery (FDMA) flap in 20.4% (n=11) cases. Other flaps used are given in Fig.1.

**Table-II:** Mechanism of injury vs time of reconstruction in the study population.

Mechanism of Injury	Time of Reconstruction (weeks)						Total

	1.00	2.00	3.00	4.00	5.00	8.00	
Blast Injuries	0 (0.00 %)	0	4 (57.1%)	1 (14.9%)	1 (14.9%)	1 (14.9%)	7 (100%)
RTA	0 (0.00 %)	1 (50%)	1 (50%)	0 (0.00 %)	0 (0.00 %)	0 (0.00 %)	2 (100%)
Electric Burns	1 (7.6%)	0 (0.00 %)	6(46.15%)	5 (38.5%)	1 (7.6%)	0 (0.00 %)	13 (100%)
Skin Malignancy Excision	2 (100%)	0 (0.00 %)	0 (0.00 %)	0 (0.00 %)	0 (0.00 %)	0 (0.00 %)	2 (100%)
Machine Injuries	7 (38.9%)	6 (33.33%)	3 (16.7%)	1 (5.55%)	1 (5.55%)	0 (0.00 %)	18 (100%)
Avulsion Injury	2 (100%)	0 (0.00 %)	0 (0.00 %)	0 (0.00 %)	0 (0.00 %)	0 (0.00 %)	2 (100%)
Contracture	7 (100%)	0 (0.00 %)	0 (0.00 %)	0 (0.00 %)	0 (0.00 %)	0 (0.00 %)	7 (100%)
Congenital	1 (100%)	0(0.00 %)	0 (0.00 %)	0 (0.00 %)	0 (0.00 %)	0 (0.00 %)	1 (100%)
Infection	0 (0.00 %)	0 (0.00 %)	0 (0.00 %)	1 (50 %)	1 (50%)	0 (0.00 %)	2 (100%)

Total	20	7	14	8	4	1	54

The commonly done flaps in digits are hetero-digital flaps and Quaba flap. Out of the total, 88.9% (n=48) reconstruction was completed in a single stage. Flap necrosis was observed in three cases (5.6%), out of which two flaps were completely lost while in one case there was insignificant partial necrosis. Surgical site infection was observed in three cases (5.6%) out of which two were local infections and in one septicemia developed. In 19 cases, bony fixation was needed, which was mostly (n=16, 84.2%) performed with k-wire fixation.

## DISCUSSION

In our study, the population sustaining hand injuries is mostly young males. Seventy five percent of our population consists of male patients. A study done in Poland showed similar results with more than 80% of their population comprising of males.15 Another study done in Mexico also showed similar distribution of population on basis of gender.6 The reason for skewed distribution towards males could be that males representation in labor industry is more as compared to females.16 Polytrauma patients also show similar trend for gender distribution.4 Most common etiology in our study population was machine injuries followed by electric burns.

Industrial and work related hand injuries are more common in low income populations.2 Burn injuries are also common in deprived population groups and usually require long follow-up with risk of recurrent contractures.16,17 As most of our patients work as labor in the industry and in workshops they present with predominance of machine injuries. One in every four patients presented with tendon injuries and 16% patients presented with amputations. Studies report similar numbers for cases presenting with tendon injuries.3,15,18

Almost half of our patients presented with no other structure involvement except for soft tissue. Although initial assessment for involvement of tendons and neurovascular element are very important, they are unlikely to be present in most cases. This is true specifically for blunt injuries. Injuries due to sharp objects are more likely to involve tendons and neurovascular structures.19 Hand injuries are complex in nature and require timely intervention by a hand surgeon.15,20 If not done so, they can lead to significant decrease in the quality of life and can severely impact upon the psychological status of the patient.7,21-23 We have given a standardized approach for soft tissue reconstruction of the hand injuries based on our experience with hand reconstruction. This will not only simplify the treatment for surgeons but will also help us in comparing outcomes of different reconstructive options used in hand.

This algorithm will also serve as a didactic tool for new hand surgeons. As data was collected from patient records, some variables that the authors wanted to study cannot be included due to presence of limited data. Many authors in the literature have described individual flaps and their techniques for wound coverage, however, further work needs to be done to assess the outcomes for each reconstructive option given in the algorithm as there is little work done on the systematic approach towards reconstruction of such defects. Other reconstructive techniques should also be explored in order to build up on current data. In this study we present our experience in management of hand injuries. We have developed an algorithm for treatment of these defects. This could be further improved by using specific outcome measures to evaluate the results of our procedures. Further studies are needed to refine this treatment algorithm with regards to outcome.

## CONCLUSION

Hand injuries commonly present to emergency department. As there are no standard guidelines for management of soft tissue defect of hand, we have presented our experience regarding such injuries. This treatment algorithm will help with the decision making process of hand reconstruction. It will also form the foundation for further work on this aspect of hand injuries.

### Authors Contribution:

**MK:** Conception, design, data gathering and data interpretation, writing, responsible for the integrity of work. **WH:** Design, Data interpretation, Writing, proofreading, literature review, responsible for integrity of work. **HU:** Data gathering, literature search, proofreading.

**NHK:** Data gathering, data interpretation, writing.
